# Bone Mineral Density and Risk of Osteoporotic Fractures in Women with Parkinson's Disease

**DOI:** 10.1155/2020/5027973

**Published:** 2020-03-25

**Authors:** Maryna Bystrytska, Vladyslav Povoroznyuk, Nataliia Grygorieva, Iryna Karaban, Nina Karasevich

**Affiliations:** ^1^Department of Clinical Physiology & Pathology of Locomotor Apparatus, SI “D. F. Chebotarev Institute of Gerontology NAMS of Ukraine”, Kyiv, Ukraine; ^2^Department of Clinical Physiology & Pathology of Extrapiramide Nervous System, SI “D. F. Chebotarev Institute of Gerontology NAMS of Ukraine”, Kyiv, Ukraine

## Abstract

Osteoporosis and Parkinson's disease (PD) are two important age-related diseases, which have an influence on pain, physical activity, disability, and mortality. The aim of this research was to study the parameters of bone mineral density (BMD), frequency, and 10-year probability of osteoporotic fractures (OFs) in females with Parkinson's disease (PD). We have examined 113 postmenopausal women aged 50–74 years old which were divided into 2 groups (I, control group (CG), *n* = 53 and II, subjects with PD, *n* = 60). Bone mineral density of lumbar spine, femoral neck, distal radius, and total body were measured, and quantity and localization of vertebral deformities were performed by the vertebral fracture assessment (VFA). Ten-year probability of OFs was assessed by Ukrainian version of FRAX®. It was established that BMD of lumbar spine, femoral neck, distal radius, and total body in PD women was reliably lower compared to CG. The frequency of OFs in PD subjects was higher compared to CG (51.7 and 11.3%, respectively) with prevalence of vertebral fractures (VFs) in women with PD (52.6% among all fractures). 47.4% of the females had combined VFs: 74.2% of VFs were in thoracic part of the spine and 73.7% were wedge ones. Ten-year probability of major OFs and hip fracture were higher in PD women compared to CG with and without BMD measurements. Inclusion of PD in the FRAX calculation increased the requirement of antiosteoporotic treatment from 5 to 28% (without additional examination) and increased the need of additional BMD measurement from 50 to 68%. Anterior/posterior vertebral height ratios (Th_8_-Th_11_) measured by VFA in PD females without confirmed vertebral deformities were lower compared to indices of CG. In conclusion, women with PD have lower BMD indices, higher rate of osteoporosis, and risk of future low-energy fractures that should be taken into account in the assessment of their osteoporosis risk and clinical management.

## 1. Introduction

Osteoporosis and Parkinson's disease (PD) are both age-related diseases which have a great impact on morbidity and mortality. Their frequency increases with age progressively and their combination can aggravate their own courses. Some recent reviews confirmed the progressive bone loss [1–5] and increased risk of the pain, disability and low-energy fractures [6–9] in females with PD; however, the exact mechanisms of osteoporosis and its consequences in PD subjects are unclear. Current studies demonstrate the lower indices of bone mineral density (BMD) in patients with PD; however, these data are contradictory and depends on sex, ethnic, and other features [1–5].

Vertebral fractures are crucial complication of systemic osteoporosis in PD patients [7, 9]. Nowadays, the important tool (vertebral fractures assessment, VFA) is widely used in routine clinical practice [10] to identify subclinical vertebral fractures and risk of further osteoporotic fractures. However, similar studies in the patients with PD are absent.

The last decade new tool for fracture risk assessment (FRAX) appeared [11]; however, its significance in patients with Parkinson's disease is unclear. Despite the fact that PD is a well-known reason of secondary osteoporosis, it is not included in list of FRAX for calculation of 10 years' probability of osteoporotic fractures and utilities of FRAX for these patients require further investigations.

The research was aimed to study the parameters of bone mineral density, frequency, and 10-year probability of osteoporotic fractures in females with Parkinson's disease.

## 2. Materials and Methods

### 2.1. Study Population

The research was conducted at the D. F. Chebotarev Institute of Gerontology NAMS Ukraine in collaboration with its two units: Department of Clinical Physiology & Pathology of Locomotor Apparatus and Department of Clinical Physiology & Pathology of Extrapiramide Nervous System. The research was approved by Ethics Committee of the Institute (19/12/2014). All subjects were recruited from 01.2015 to 12.2016 and signed the informed consent for participation in this study and/or treatment in institution clinic.

We used a cross-sectional case-control research design and examined 113 postmenopausal women aged 50–74 years old which were divided into two groups: Group I, females without PD and any other conditions or illnesses which can have the influence on bone state and metabolism (control group, *n* = 53) and Group II, subjects with PD (*n* = 60). Patients of Group I were examined at the Department of Physiology and Pathology of Locomotor Apparatus without being admitted to a hospital. All patients of Group II were hospitalized at the Department of Clinical Physiology & Pathology of Extrapiramide Nervous System of D. F. Chebotarev Institute of Gerontology NAMS of Ukraine for regular (one time a year) complex neurological examination and treatment correction.

The diagnosis of PD in women of Group II was established according to the criteria of the Bank of the Brain of the British Society of Parkinson's Disease, and its stages were assessed by the M. Hoehn and M. Yahr (H and Y) classification [12]. All patients with PD were at levodopa therapy. All examined subjects were not previously examined for osteoporosis by the dual-energy X-ray absorptiometry (DXA) method and did not receive any drugs for its prevention and treatment (including calcium and vitamin D, etc.).

The exclusion criteria for both groups were any chronic diseases, which affect the bone metabolism, mental diseases, recent surgery, and glucocorticoids use. Additionally, we excluded patients with 4-5 stages of PD according to the H and Y classification, pronounced tremor, and other severe motor and postural disturbances (camptocormia, scoliosis, and Pisa syndrome) that interfere with conducting and evaluating of dual-energy X-ray absorptiometry (DXA) and interpretation of study results.

The mean age of PD onset in subjects of Group II was 57.5 ± 8.2 years, the mean duration of PD was 7.2 ± 4.2 years, and the dose of levodopa therapy was 429 ± 222 mg/d. Mean parameters of Unified Parkinson's Disease Rating Scale [13] in PD women consisted of UPDRS I (mentation, behavior, and mood) subscale 1.76 ± 2.02 un., UPDRS II (activities of daily living) subscale 13.38 ± 5.58 un., UPDRS III (motor) subscale 38.45 ± 10.14 un., total count 53.60 ± 14.40 un.

### 2.2. Assessments

#### 2.2.1. Questionnaire

Fracture rate was studied using questionnaire about quantity and localization of previous fractures, their reasons, and outcomes. The investigator confirmed the fracture history according to the medical source.

Ten-year probability of major osteoporotic fractures (MOF) and hip fractures (HF) was performed by FRAX® which is a well-known calculator for assessment of fracture risk [10]. We used Ukrainian version for fractures risk assessment [14]. FRAX® model accounts for most reverent risk factors for osteoporotic fractures, including different causes of secondary osteoporosis (insulin-dependent diabetes mellitus, osteogenesis imperfecta in adults, untreated long-standing hyperthyroidism, hypogonadism or premature menopause, chronic malnutrition, or malabsorption and chronic liver disease). Parkinson's disease is not included in this list; however, nowadays, it is a confirmed risk factor for osteoporosis. We calculated FRAX indices (FRAX-MOF and FRAX-HF for MOF and HF, respectively) using three different approaches (six indices). The first one was by including BMI parameters (without BMD), the second approach assumed the use of BMD index, and the third one was calculated without BMD, but with inclusion of PD in the model as a cause of secondary osteoporosis. Additionally, we have compared the FRAX-MOF indices (calculated without BMD, two parameters) with reference data of Ukrainian model [15].

#### 2.2.2. Bone Mineral Density and Vertebral Fractures Assessments

Bone mineral density of lumbar spine, femoral neck, distal radius, and total body and *T*- and *Z*-scores (which reflects the comparison with healthy young (20 years) adults and age-matched population, accordingly) were measured using the DXA method (Prodigy, GEHC Lunar, Madison, WI, USA). Interpretation of DXA results for postmenopausal women was conducted according to the International Society for Clinical Densitometry (ISCD) recommendations [16] according to the lowest *T*-score at lumbar spine or femoral neck/total hip (normal bone (*T*-score > −1.0 SD), osteopenia (≤(−1.0) *T*-score > (−2.5), SD and osteoporosis (*T*-score ≤ −2.5 SD).

The investigator confirmed the fracture history according to medical source. Vertebral fractures were affirmed by vertebral fracture assessment (VFA) which was performed additionally to BMD measurements by DXA on the lateral VFA images of the spine [16–18]. During this procedure, the patient was in supine position and a cushion supported the knees. The DXA software generated six points on each vertebral endplate. The dedicated technician manually adjusted them and calculated the anterior, middle, and posterior vertebral height (*T*_4_-*L*_4_) and ratios between them using a standardized protocol with the detector centralized on Th_7_ (for the thoracic spine) and on *L*_3_ (for the lumbar spine). Following this quantitative evaluation, the software used the criteria of Genant's classification [19] for vertebral fractures.

#### 2.2.3. Data Analysis

We performed the statistical analysis using the package of “Statistica 11.0” Copyright© StatSoft, Inc. 1984–2011 software (Serial Number: STA999K347150-W). We used the method of descriptive statistics; the distribution of all variables was tested using the Shapiro-Wilk's *W* test. The parameters are represented at mean (*M*) ± standard deviation (SD) in case of parametric distribution and median (Me) and quartiles [Q25–Q75] (nonparametric one). In order to have statistical power of 80% with a two-sided 5% level of significance (*P* < 0.05), we calculated the size of research sample which was required to detect the differences of studied parameters between two groups. Comparison of two independent groups was performed by Student's *t*-test, Mann-Whitney *U* test, or *χ*_с_^2^ test, accordingly.

## 3. Results

### 3.1. Participants

We have studied BMD, frequency, and 10-year probability of osteoporotic fractures in 113 postmenopausal females aged 50–74 years who were divided into groups concerning PD presence. The women from both groups did not differ significantly in parameters of age (for control group and women with PD, it was 65.17 ± 6.50 and 66.40 ± 8.21 years, respectively, *t* = 0.87; *P*=0.38, accordingly) and main anthropometrical parameters (height: 1.58 ± 0.05 and 1.59 ± 0.07 m, *t* = 1.20; *P*=0.23; weight: 72.58 ± 10.58 and 70.58 ± 13.20 kg, *t* = 0.88; *P*=0.38; body mass index (BMI): 29.07 ± 4.43 and 27.32 ± 5.70, kg/m^2^, *t* = 1.81; *P*=0.07, accordingly). Also they did not differ in indices of menopause age (49.36 ± 4.62 and 49.50 ± 3.72 years, *t* = 0.15; *P*=0.88) and duration of postmenopausal period (15.0 [7.0–15.0] and 16.0 [10.0–22.0] years, *Z* = 1.22; *P*=0.21 at Groups I and II, respectively).

### 3.2. Bone Mineral Density in Women with Parkinson's Disease

Analysis of BMD indices revealed that all DXA parameters were significantly lower in PD females compared to similar indices in control group regardless of the localization of the measurement. Thus, lumbar spine BMD parameters were 0.97 ± 0.32 and 1.09 ± 0.13 g/cm^2^, respectively (*t* = 3.64; *P* < 0.001), femoral neck BMD indices were 0.78 ± 0.13 and 0.86 ± 0.11 g/cm^2^ (*t* = 4.28; *P* < 0.001), and total body BMD indices were 1.02 ± 0.13 and 1.09 ± 0.08 g/cm^2^, accordingly (*t* = 3.20; *P* < 0.01, Table 1).

### 3.3. Frequency of Osteoporosis and Low-Energy Fractures in Subjects with Parkinson's Disease

Osteoporosis in PD women was registered more frequently than in control subjects (*χ*_с_^2^ = 20.1, confidential interval (CI): 22.6–52.4, *P* < 0.001). Analysis of distribution according to the bone deterioration (osteopenia and osteoporosis) in PD women demonstrated that 48.3% of females had osteoporosis, 38.3% had osteopenia, and 13.41% had normal BMD indices compared to 9.4, 64.2, and 26.4%, respectively, in control group.

Analysis of fracture frequency in females depending on PD presence showed that 11.3% of women from Group I and 51.7% from Group II had low-energy fractures (*χ*^2^ = 20.7; 95% CI: 24.7–54.0; *P* < 0.001).

One subject (1.7%) with PD had previous bilateral hip fracture, 31.7% of PD subjects had vertebral fractures (61.3% from all fractures in these groups), 11.7% had distal forearm fractures, and 8.3% had other nonvertebral fractures. All females from control group had nonvertebral fractures; half of them had distal forearm fractures.

### 3.4. Evaluation of Vertebral Deformities by Vertebral Fractures Assessment Method in Women with Parkinson's Disease

**Figure 1:**
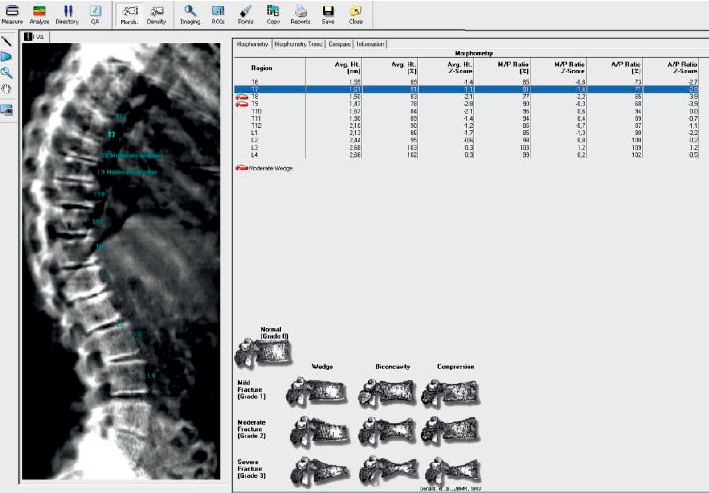
Image of vertebral fracture assessment in patient with Parkinson's disease.

Vertebral deformities (VD) were confirmed in 19 females with PD: 10 (52.6%) of them had 1 VD and 9 (47.4%) women had two or more vertebral deformities (VDs) (Figure 1).

Analysis of VDs distribution according to their level in spine (thoracic or lumbar ones) showed that most patients (13, 68.4%) had VDs in thoracic spine. Only three females (15.8%) had VDs in lumbar spine and another three (15.8%) had combined (thoracic and lumbar spine) VDs. Among patients with VDs, two subjects had Th_6_ VD, three had Th_7_ VD, two women had Th_8_ VD, one patient had Th_9_ VD, four females had Th_10_ VD, and one woman had Th_11_ VD. Five patients had combined VDs, four of them had Th_12_ та *L*_1_ VDs, and one subject had combined *L*_2_, *L*_3_, and *L*_4_ VDs.

Analysis of VD types has shown that wedge fractures of different grades were the most common (73.7%) VDs. Only three (15.8%) patients had crush VDs and another two (10.5%) had biconcave VDs.

**Figure 2:**
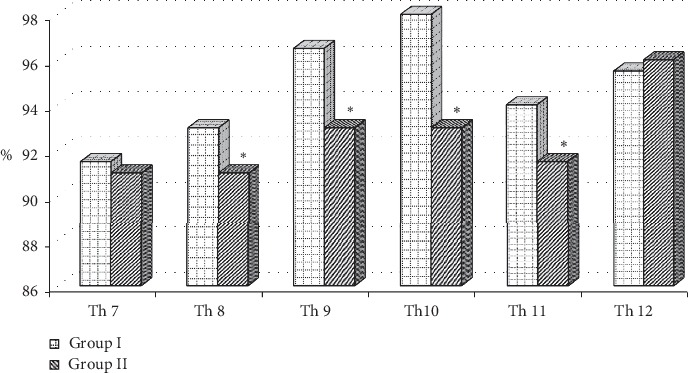
Ratios of the anterior to the posterior vertebral bodies height in the thoracic spine in women depending on Parkinson's disease presence, %. Group I: control group; Group II: females with PD. ^*∗*^Significant differences (*P* < 0.05) between the indices. Data (%) presented in medians.

After excluding from analysis confirmed VDs, we performed the additional assessments of parameters of anterior, middle, and posterior heights of vertebral bodies and revealed that PD women had significantly lower parameters of anterior vertebral body height compared to females of control group which consisted of Th_8_ (1.63 [1.53–1.72] and 1.66 [1.59–1.76] сm), Th_9_ (1.73 [1.61–1.82] and 1.77 [1.68–1.87] сm), Th_10_ (1.86 [1.71–1.97] and 1.91 [1.81–2.01] cm), Th_11_ (1.95 [1.82–2.00] and 2.01 [1.90–2.11] cm); *P* < 0.05 for all indices. Also, we found the reliably lower parameters of anterior/posterior height ratios in PD females compared to control group at the level of Th_8,_ Th_9,_ Th_10_, and Th_11_ (Figure 2). However, we did not establish the significant differences between parameters of middle and posterior heights and their height ratios in females depending on PD presence in subjects without VDs.

Among patients with previous fractures (*n* = 31), osteoporosis was established in 22 subjects (70.9%), and osteopenia was established in eight women (25.8%). Only one female with a previous fracture (3.3%) had normal BMD index; however, she confirmed a previous low-energy distal radius fracture.

Analysis of the frequency of bone state disturbances (osteopenia or osteoporosis) in patients with VDs demonstrated that 31.6% of them had osteopenia and 68.4% had osteoporosis at lumbar spine (*L*_1_–*L*_4_).

### 3.5. Indices of FRAX in Females with Parkinson's Disease

Assessment of 10-year probability of MOF and HF using Ukrainian version of FRAX revealed the significant differences between the groups using different types of calculation (Table 2).

Despite the fact that all subjects from both groups did not have rheumatoid arthritis, glucocorticoids use (which included in FRAX), and other reasons of secondary osteoporosis, 8% of subject from control group and 13% of patients of PD group have a family history of hip fractures. According to FRAX-MOF indices (calculated with BMI without BMD), 5% of patients with PD required the antiosteoporotic treatment without BMD measurement (high fractures risk), 50% needed additional DXA examination (medium fractures risk), and 45% did not require any additional examination for fractures risk assessment and antiosteoporotic treatment (low fractures risk). The correspondent parameters in control group were 0, 40, and 60%. The inclusion of PD in FRAX calculation as a reason of secondary osteoporosis increased the quantity of patients which required antiosteoporotic treatment (28%) or additional DXA examination (68%) and decreased the number of subjects who did not need any additional assessment or treatment (2%).

## 4. Discussion

Systemic osteoporosis and Parkinson's disease are two important age-related diseases, which have great influence on restriction of physical activity, disability, and mortality [1–3]. Osteoporosis makes the course of the disease worse in women of older age groups. Each of these diseases has its own particularities in older women; however, their combination can have a great impact on life duration and quality.

Recent reviews demonstrated [3–8] that female gender, disease duration and severity, old age, and low BMI are related to osteoporosis, whereas rigidity, bradykinesia, and postural instability (but not tremor) predict falls and increase the risk of osteoporotic fractures.

Some authors showed that females and males with PD had significantly lower BMD values at femoral neck, whereas only female patients showed reliable differences in the lumbar spine BMD compared to subjects of control group [20]. In contrast, Turkish research [21] demonstrated that BMD indices of lumbar spine and femoral neck were lower compared to the PD patients, irrespective of gender. In this study, we showed that parameters of BMD in different regions of skeleton are significantly lower in PD women compared to subjects from control group and frequency of osteoporosis is higher. However, earlier, we [22] demonstrated the increased rate of osteoporosis in Ukrainian PD men with reliably lower total radius and total body BMD and without any significant differences in the lumbar spine and femoral neck. It confirms the influence of gender on bone loss in subjects with PD and requires the future studies.

Nowadays, the relationship between PD and risk of osteoporotic fracture yielded inconsistent results [6–8, 23]. Recent meta-analysis of prospective studies, which was performed to explore the association between PD and fracture risk (six studies with 69387 participants) [6], found out that PD patients had an increased risk of fracture compared to control subjects (hazard ratio (HR) = 2.66, 95% CI: 2.10–3.36). Subgroup analysis showed the similar risk in males and females. Another recent systematic review and meta-analysis of published studies concerning association between PD and risk of hip fracture (13 studies with 564947 participants), which was by conducted by Hosseinzadeh et al. [8], demonstrated that PD is associated with the risk of hip fracture (HR overall = 3.13, 95% CI: 2.53–3.87); however, it is greater in women than in men.

Vertebral fractures are ones of the most dangerous osteoporotic fractures. Usually, they are divided into three subtypes: wedge, biconcave, and crush fractures. The first ones are the most common, accounting for more than 50% of all VDs. These fractures are characterized by compression of the anterior part of the vertebrae. Biconcave fractures are the second most common, accounting for approximately 17% of all VDs, which are characterized by compression of middle part of the vertebral body. The crush fractures are the least common VDs which account for only 13% of VDs and are characterized by compression of the all portions of vertebrae [24].

Analysis of osteoporotic fractures rate in PD subjects has shown their higher frequency in PD females compared to control (51.7 and 11.3%, respectively) with prevalence of vertebral fractures in women with PD (52.6% among all fractures), whereas we did not reveal vertebral fractures in control group. 47.4% of the patients had combined vertebral fractures; 74.2% of the vertebral fractures were in thoracic part of the spine and 73.7% were wedge ones. High rate of vertebral fractures in PD women can be related to vertebral pain syndrome and limitation of physical activity. They are negative factors for future osteoporotic fractures that require future study.

Vertebral fracture assessment is one of the substantial additional methods of bone tissue appraisal in patients with osteoporosis [1, 16–18]. According to Genant's classification [19] which is based on the vertebral shape with respect to vertebral height loss and included in DXA software, the loss of >25% of vertebral height (anterior, posterior, and/or middle) is confirmed as moderate and severe VDs (grades 2 and 3). However, grade 1 supposes <25% loss of vertebral height and is named as mild VD. Their presence and quantity can be significant for disease progression; however, we did not find fitting studies in the literature which assess the possibility of VFA in fracture risk assessment in PD patients. However, our research revealed the lower parameters of anterior/posterior height ratios (Th_8,_ Th_9,_ Th_10_, and Th_11_) in PD females compared to control group even after excluding the VDs from the analysis.

The reduction of the anterior parts of the vertebrae in the middle and lower parts of thoracic spine may be a component for formation of the specific posture in PD patients. Spinal deformities in PD subjects can be related to muscle function changes, pain syndrome, and so on [23]. We are agreed that elevated muscle tone has a great impact on the formation of a spine distortion in PD subjects. However, 68.42% of patients without VDs had confirmed osteoporosis at lumbar spine (*L*_1_–*L*_4_) (31.6% women had osteopenia), which can have an influence on forming of the vertebral pain and posture in PD patients.

Additionally, the high prevalence of wedge deformities in the thoracic spine may result from muscle rigidity with the formation of a specific posture in patients with Parkinson's disease, antecollis. Probably deformities of the thoracic vertebrae contribute to the progression of postural disorders in patients with Parkinson's disease, although we did not include the patients with major postural disturbances (camptocormia, scoliosis, and Pisa syndrome) which have an impact on DXA performing and interpretation. This issue requires future studies.

Current literature data confirm that PD is an important risk factor for fragility fractures. Lee et al. [9] found that prevalence of PD in subjects with osteoporosis consists of 12.8–14.4% regardless of sex or age. More than 95% of patients with PD and osteoporosis are older than 60 years old and women prevail over the men. However, nowadays, PD is not included in list of secondary osteoporosis in FRAX model [25] that can have a negative influence in assessment of osteoporotic fractures risk. The FRAX possibility in fracture risk assessment was studied in limited researches [26] and requires future investigations.

In this study we used the Ukrainian model of FRAX [14], which was developed a couple of years ago, and used three different approaches in our calculation (with BMI and BMD and including PD as a cause of secondary osteoporosis). We revealed the increased 10-year probabilities of MOF and HF in patients with PD using all approaches despite the absence of significant differences in age, height, and weight parameters and absence of rheumatoid arthritis and glucocorticoids use (which was included in FRAX) in all subjects from both groups. Also, we found that despite of the significant differences in FRAX indices calculated without or with BMD, small quantity of patients with PD (5%) required antiosteoporotic treatment (despite the fact that 51.7% of them have previous fractures) when PD was not included as a reason of secondary osteoporosis. Inclusion of this risk factor in this model increased the requirement of antiosteoporotic treatment from 5 to 28% (without any additional examination) and increased the requirement in additional DXA examination from 50 to 68%.

The limitations of our study were being of a cross-sectional design, the sample size, and inclusion of only patients with second and third stages of disease (according to the H and Y) without severe motor and postural disturbances. Further large-scale longitudinal studies are required to find the association between PD and osteoporosis more fully for determining bone-safe strategy for women with PD.

## 5. Conclusion

The results of our study demonstrated the significant difference between the BMD indices at lumbar spine, femoral neck, distal radius, and total body in PD females compared to control group. The frequency of osteoporotic fractures in PD subjects was higher compared to women of control group (51.7 and 11.3%, respectively) with prevalence of vertebral fractures in females with PD (52.6% among all fractures). 47.4% of the females have combined VFs; 74.2% of vertebral fractures are in thoracic part of the spine and 73.7% are wedge ones. FRAX indices for major osteoporotic fractures and hip fracture were higher in PD women compared to control group. Inclusion of PD in the FRAX calculation increased the requirement of antiosteoporotic treatment from 5 to 28% (without additional examination) and increased the need of additional BMD measurement from 50 to 68%. The anterior/posterior height ratios (Th_8_–Th_11_) measured by VFA in PD females without confirmed vertebral deformities were significantly lower compared to indices of control group. Neurologists should pay attention to the osteoporosis risk in PD women, and adequate preventive strategies should be taken in order to maintain bone health and decrease the risk of the future fractures due to osteoporosis.

## Figures and Tables

**Table 1:** Bone mineral density indices in females depending on PD presence (*M* ± SD).

Index/group	Group I	Group II	*t*	*P*
*L*_1_-*L*_4_				
BMD, g/cm^2^	1.09 ± 0.13	0.97 ± 0.32	3.64	<0.001
*T*-score, SD	−0.74 ± 1.12	−1.70 ± 1.59	3.63	<0.001
*Z*-score, SD	0.39 ± 1.02	−0.45 ± 1.38	3.58	<0.001

Femoral neck				
BMD, g/cm^2^	0.86 ± 0.11	0.78 ± 0.13	4.28	<0.001
*T*-score, SD	−1.17 ± 0.77	−1.81 ± 0.96	3.83	<0.001
*Z*-score, SD	0.13 ± 0.67	−0.42 ± 0.81	3.87	<0.001

Total hip				
BMD, g/cm^2^	0.94 ± 0.11	0.86 ± 0.16	3.11	<0.01
*T*-score, SD	−0.53 ± 0.89	−1.19 ± 1.26	3.12	<0.01
*Z*-score, SD	0.49 ± 0.75	−0.07 ± 1.05	3.17	<0.01

Total radius				
BMD, g/cm^2^	0.77 ± 0.08	0.65 ± 0.16	4.23	<0.001
*T*-score, SD	−1.31 ± 1.14	−2.29 ± 1.53	3.50	<0.001
*Z*-score, SD	−0.07 ± 1.09	−0.83 ± 1.29	3.23	<0.01

Total body				
BMD, g/cm^2^	1.09 ± 0.08	1.02 ± 0.13	3.20	<0.01
*T*-score, SD	−0.42 ± 1.01	−1.18 ± 1.47	3.15	<0.01
*Z*-score, SD	0.34 ± 0.88	−0.25 ± 1.11	3.06	<0.01

Group I: control group; Group II: females with Parkinson's disease; *t* and *P*: differences between the indices in women of both groups (Student's *t*-test).

**Table 2:** Parameters of 10-year probability of osteoporotic fractures in subjects depending on presence of Parkinson's disease (Me [25Q–75Q]), %.

Index/group	Group I	Group II	*Z*	*P*
FRAX-MOF^1^	4.1 [3.4–5.4]	5.1 [3.6–8.1]	2.3	<0.01
FRAX-HF^1^	0.9 [0.6–1.5]	1.6 [0.5–2.4]	2.2	<0.01
FRAX-MOF^2^	3.8 [3.3–5.4]	5.2 [3.9–9.8]	4.1	<0.001
FRAX-HF^2^	0.6 [0.4–1.2]	1.3 [0.6–3.5]	4.0	<0.001
FRAX-MOF^3^	4.1 [3.4–5.4]	7.4 [4.9–11.0]	6.0	<0.001
FRAX-HF^3^	0.9 [0.6–1.5]	2.8 [0.9–4.3]	5.0	<0.001

Group I: control group; Group II: females with Parkinson's disease. ^1^Calculated with body mass index. ^2^Calculated with BMD. ^3^Calculated without BMD and including PD as a reason of secondary osteoporosis; *Z* and *P*: differences between the indices in women of both groups (Mann-Whitney *U* test).

## Data Availability

All data used to support the results of this study are stored at and available from the corresponding author upon request.
